# Supplementary material for: Why managing sciatica is difficult: patients’ experiences of an NHS sciatica pathway. A qualitative, interpretative study
Source: BMJ Open. 2020 Jun 11;10(6):e037157. doi: 10.1136/bmjopen-2020-037157 (PMC7295411; doi:10.1136/bmjopen-2020-037157)
Supplement: Author's manuscript [file bmjopen-2020-037157.draft_revisions.pdf]

BMJ Open

BMJ Open is committed to open peer review. As part of this commitment we make the peer review history of every article we publish publicly available.

When an article is published we post the peer reviewers' comments and the authors' responses online. We also post the versions of the paper that were used during peer review. These are the versions that the peer review comments apply to.

The versions of the paper that follow are the versions that were submitted during the peer review process. They are not the versions of record or the final published versions. They should not be cited or distributed as the published version of this manuscript.

BMJ Open is an open access journal and the full, final, typeset and author-corrected version of record of the manuscript is available on our site with no access controls, subscription charges or pay-per-view fees (http://bmjopen.bmj.com).

If you have any questions on BMJ Open's open peer review process please email info.bmjopen@bmj.com

Why managing sciatica is difficult: patients' experiences of a new national sciatica pathway. A qualitative interpretative study.

Journal:	*BMJ Open*
Manuscript ID	bmjopen-2020-037157
Article Type:	Original research
Date Submitted by the Author:	21-Jan-2020
Complete List of Authors:	Ryan, Clare; Solent NHS Trust, Physiotherapy Service; University of Southampton, School of Health Sciences<br>Pope, Catherine; University of Oxford, Nuffield Department of Primary Care Health Sciences<br>Roberts, Lisa; University of Southampton, School of Health Sciences; University Hospital Southampton NHS Foundation Trust, Therapy Services Department
Keywords:	Back pain < ORTHOPAEDIC & TRAUMA SURGERY, Musculoskeletal disorders < ORTHOPAEDIC & TRAUMA SURGERY, Adult orthopaedics < ORTHOPAEDIC & TRAUMA SURGERY, QUALITATIVE RESEARCH, Quality in health care < HEALTH SERVICES ADMINISTRATION & MANAGEMENT, Protocols & guidelines < HEALTH SERVICES ADMINISTRATION & MANAGEMENT

SCHOLARONE™ Manuscripts

I, the Submitting Author has the right to grant and does grant on behalf of all authors of the Work (as defined in the below author licence), an exclusive licence and/or a non-exclusive licence for contributions from authors who are: i) UK Crown employees; ii) where BMJ has agreed a CC-BY licence shall apply, and/or iii) in accordance with the terms applicable for US Federal Government officers or employees acting as part of their official duties; on a worldwide, perpetual, irrevocable, royalty-free basis to BMJ Publishing Group Ltd ("BMJ") its licensees and where the relevant Journal is co-owned by BMJ to the co-owners of the Journal, to publish the Work in this journal and any other BMJ products and to exploit all rights, as set out in our licence.

The Submitting Author accepts and understands that any supply made under these terms is made by BMJ to the Submitting Author unless you are acting as an employee on behalf of your employer or a postgraduate student of an affiliated institution which is paying any applicable article publishing charge ("APC") for Open Access articles. Where the Submitting Author wishes to make the Work available on an Open Access basis (and intends to pay the relevant APC), the terms of reuse of such Open Access shall be governed by a Creative Commons licence – details of these licences and which Creative Commons licence will apply to this Work are set out in our licence referred to above.

Other than as permitted in any relevant BMJ Author's Self Archiving Policies, I confirm this Work has not been accepted for publication elsewhere, is not being considered for publication elsewhere and does not duplicate material already published. I confirm all authors consent to publication of this Work and authorise the granting of this licence.

For peer review only

Clare Ryan[1&2] (ORCID ID: 0000-0002-3555-8624),

Professor Catherine Pope[3] (ORCID ID: 0000 0002 8935 6702)

Professor Lisa Roberts[2&4] (ORCHID ID: 0000-0003-2662-6696)

WHY MANAGING SCIATICA IS DIFFICULT: PATIENTS' EXPERIENCES OF A NEW NATIONAL SCIATICA PATHWAY. A QUALITATIVE, INTERPRETATIVE STUDY.

[1]Physiotherapy Service, Solent NHS Trust, Portsmouth, UK.

[2]School of Health Sciences, University of Southampton, UK.

[3]Nuffield Department of Primary Care Health Sciences, University of Oxford, UK.

[4] Therapy Services Department, University Hospital Southampton NHS Foundation Trust, Southampton, UK.

Corresponding author: Clare Ryan, Physiotherapy Service, Solent NHS Trust, St Mary's Community Health Campus, Block A First Floor, Milton Road, Portsmouth, UK, PO3 6AD (Tel: +44 7531 332325). Email: Clare.ryan1@nhs.net

Word Count: 3,817

For peer review only

ABSTRACT

Objectives: Amid a political agenda for integrated, high-value care, the UK is implementing its Low Back and Radicular Pain Pathway. To align care with need, it is imperative to understand the patients' perspective. The purpose of this study was therefore, to explore how people experience being managed for sciatica within an NHS care-pathway.

Design: Qualitative interpretative study.

Setting: Musculoskeletal Service in an NHS, Primary Care Trust, UK.

Participants: The sample comprised 14 people aged ≥18years with a clinical presentation of sciatica, who were currently under the care of a specialist physiotherapist (the specialist spinal triage practitioner), had undergone investigations (magnetic resonance imaging) and received the results within the past six weeks. People were excluded if they had previously undergone spinal surgery or if the suspected cause of symptoms was cauda equina syndrome or sinister pathology. Participants were sampled purposively for variation in age and gender. Data were collected using individual semi-structured interviews (duration: 38-117 minutes; median: 82.6 minutes), which were audio-recorded and transcribed verbatim. Data were analysed thematically.

Results: A series of problems with the pathway (insufficient transparency and information; clinician-led decisions; standardised management; restricted access to specialist care; and a lack of collaboration between services) made it difficult for patients to access the management they perceived necessary. Patients were therefore required to be independent and proactive, or to have agency. Agency was, however, difficult to enact (due to the impact of sciatica and because patients lacked the necessary skills, funds and support) and together with the pathway issues, this negated patients' capability to manage sciatica.

Conclusions: This novel paper explores how patients with sciatica experience the process of being managed within a care pathway. Whilst highlighting the need to align with recommended best practice, it shows the need to be more person-centred and to support and empower patient agency.

Keywords: Agency; Care-Pathway; Patient experience; Qualitative Research; Sciatica; Treatment Burden.

ClinicalTrials.gov reference: UOS-2307-CR

ARTICLE SUMMARY

Strengths and limitations of this study

- To our knowledge, this paper is unique in exploring how patients with sciatica experience the *process* of being managed within a care-pathway.
- Although this is a small, single site study it is rich in detail and has conceptual transferability to similar services at an early stage of pathway implementation managing patients who have not improved with conservative treatment and whose symptoms are sufficient to require investigation.
- Limitations include not undertaking iterative theoretical sampling during analysis and interviewing some participants before they had completed their management; these issues occurred as the methods were determined by the requirements of the original study.

For peer review only

INTRODUCTION

Sciatica is a common form of low back pain characterised by pain, altered sensation and/or weakness in the leg and results from a combination of compression, inflammation and/or sensitisation of a lumbar/sacral nerve root (1). The most common causes are lumbar disc-herniation and stenosis, and rarely, sinister pathology (such as cancer); however, it is not always possible to identify a structural cause(2). Sciatica prevalence estimates vary widely, from 1.2% to 43%, reflecting the use of different diagnostic criteria and sampling methods(3). Whilst many people will recover from sciatica within 12 weeks, around 30% are likely to have ongoing pain after a year(4). Patients report that sciatica can be severe and incapacitating and result in marked limitation to daily activities(5). Furthermore, it is well established that the presence of leg pain, compared to low back pain alone, adversely affects symptom severity, disability, absence from work and outcome(6).

Over the past decade, in the UK and the developed world, there has been a political agenda for healthcare that is integrated (collaborative and responsive) and high-value (aligns with best practice) (7). Reflecting these agendas, and to facilitate implementation of National Institute of Health and Care Excellence (NICE) guidance(8), a UK Low Back and Radicular Pain Pathway (9) has been developed. This pathway recommends that patients with sciatica are initially managed within primary care, with medication and/or physiotherapy. For those who fail to improve, and who have severe pain, assessment by a specialist (spinal triage) practitioner is recommended to consider management options include investigations. In the UK this role is commonly, though not exclusively, performed by advanced practice physiotherapists(9). If, through investigations such as magnetic resonance imaging, a concordant structural cause is identified, referral for an epidural injection or surgery may be offered. When no concordant structural cause is identified, or the patient wishes to avoid invasive intervention, the recommended approach is a combined physical and psychological programme. Achieving integrated, high-value care is particularly important for patients with sciatica who commonly cross traditional boundaries of care to access management from a range of health-professionals. Understanding patients' experiences of being managed for sciatica can inform the provision of care. Four qualitative UK based studies(10-13) with a total of 75 patients have provided some insight into patients' beliefs, expectations, and experiences of components of the conservative management of sciatica. However, to understand what it is like to be managed within a care-pathway, further research is required. This paper therefore addresses the question, 'what are patients' experiences of being managed within an NHS sciatica care-pathway?'

METHODS

The data for this paper were collected as part of a wider study about patients' experiences of investigations for sciatica(12). Ethical approval was gained from the South-West Ethics Committee in September 2015 (15/SW/0247). This paper is reported in line with the COREQ guidelines.

Study design

This was an interpretative, qualitative research study, a methodology consistent with the aim of exploring peoples' experiences.

For peer review only - http://bmjopen.bmj.com/site/about/guidelines.xhtml

Setting

The setting was an outpatient physiotherapy service of a UK, NHS, primary care Trust. This service was selected as an early adopter of the new national pathway and one with experienced specialist spinal triage practitioners.

Participants and recruitment

The sample comprised people aged ≥18years with a clinical presentation of sciatica, who were currently under the care of a specialist-physiotherapist (the specialist-spinal-triage-practitioner), had undergone investigations (magnetic resonance imaging (MRI)) and received the results within the past six weeks. Participants were therefore recruited at a key point in their pathway-management, when they had failed initial conservative management, had undergone investigations to determine whether a concordant structural cause could be identified, and had discussed with a specialist-physiotherapist the most appropriate next step.

The study was designed to recruit 12-15 participants. No claims were made to reach data saturation, as each person's experience of a phenomenon is unique. However, aligning with qualitative research recommendations(14, 15), this number was considered enough to enable a rich, detailed analysis of this defined, relatively homogenous group, whilst providing sufficient information to answer the research question, and sufficient variation within the sample to enhance transferability.

The clinical diagnosis of sciatica was made by the patient's specialist physiotherapist using information from their clinical assessment and based on diagnostic criteria (Fig. 1) reflecting those used in practice(16). The patient's radiological findings did not inform this diagnosis. The specialist physiotherapists were clinicians with ≥ 10 years musculoskeletal experience, who undertook specialist spinal training ≥ four times a year. We used purposive sampling to gain representation across age, sex and duration of symptoms, and recruited 14 participants, sufficient to enable rich, detailed analysis.

Fig. 1 Criteria for diagnosing sciatica(16)

Potential participants were approached about participating in the study, by their specialist physiotherapist, when they attended for their investigation results. Those interested were provided with verbal and written information about the study. With their written consent, the researcher made contact to arrange an interview date. To increase the homogeneity of the sample, people were excluded if: they had previously undergone spinal surgery; the suspected cause of symptoms was sinister pathology or cauda equina syndrome; they were unable to communicate without the assistance of an interpreter; they lacked capacity to provide consent; or the researcher had treated them in a previous episode of back pain, to minimise bias.

Data collection

The lead author (CR) a female specialist physiotherapist and MRes student (with prior experience and training in conducting qualitative interviews) collected data between October 2015 and May 2016[1] using in-depth, individual, face-

[1] Subsequent to the release of the initial National Low Back and Radicular Pain Pathway guidance in 2014

For peer review only - http://bmjopen.bmj.com/site/about/guidelines.xhtml

to-face, semi-structured interviews. This method was selected to facilitate rapport and enable emergent issues to be explored. To minimise the influence of the researcher's position, CR introduced herself as a researcher, and hosted the interviews in an individual room within the hospital, away from the physiotherapy department.

A topic guide was used as a starting point for discussion, however, interviews followed the participant's lead, with emergent issues being explored and, where indicated, incorporated into subsequent interviews. Fig. 2 details the key questions used to explore patients' experience of the pathway. Minimal facilitation was used to prevent 'leading'. At the start of data collection, CR conducted pilot interviews with two participants to provide face and content validity for the questions asked. As the content and key wording of questions were substantially unchanged, the data from these participants were included in the study. The interviews were audio-recorded, transcribed verbatim, and pseudonyms were used to maintain anonymity. We did not ask participants to validate the transcripts or findings as the usefulness of this strategy is contested(17).

Fig. 2 Topic guide

Patient and public involvement

The question addressed by this paper directly resulted from discussion with the 14 participants. Patients were not involved in data collection. Informal discussions with a series of patients, and engagement with the public during 3 dissemination events, aided the interpretation of findings.

Data analysis

We analysed the data manually, thematically and iteratively, based on the method of Braun and Clarke(18). Thematic analysis has three stages: (i) line-by-line coding, (ii) developing descriptive themes and (iii) generating analytical themes. Firstly, we explored the data on its own terms, using inductive line-by-line coding. The data relevant to the research question were then identified, explored through selective and axial coding, and initial concepts were identified. We then explored the constituent parts, relationships and potential explanations for concepts, moving back and forth between the raw data, key concepts and relevant clinical, theoretical and policy literature. We used charts to manage the data, to facilitate comparison within and between cases, and to ensure that analysis remained rooted in the data(19). We included variation and complexity and used analytical and reflexive memos to facilitate a deeper understanding(19). Each of the authors contributed to analysis; CR identified the initial codes and concepts, which CP and LR, expert qualitative researchers, interrogated to refine their scope; relevance; constituent parts; relationships; and explanatory potential. Involving three analysts with different professional backgrounds aided the rigor of analysis and provided a check to minimise potential bias, assumptions and data selection.

RESULTS

The sample comprised fourteen participants; aged 34-81 years (median 61 years); eight participants were female. Participants described a three-month to nine-year (median thirteen months) duration of symptoms. By the time of their interview, half of participants had experienced improvement in their symptoms, including 5 with significant improvement. Participant characteristics are detailed in Table 1. Four additional people were identified but not included

For peer review only - http://bmjopen.bmj.com/site/about/guidelines.xhtml

as they did not meet inclusion criteria (n=1); did not wish to participate (n=1); attended on the wrong day (n=1); or could not be contacted (n=1). Interviews ranged in length from 38-117 minutes (median 82.6 minutes).

For peer review only

For peer review only - http://bmjopen.bmj.com/site/about/guidelines.xhtml

Table 1 Participant characteristics(5, 12)

**Partici pant age and sex**	**Work status and occupation**	**Symptoms**	**Symp tom dura tion**	**Neurological findings**	**Pathway management for this episode of sciatica**	**MRI find ings***	**Likely next step**
**Julia 63yrs Female**	Part time office worker	Leg pain, altered sensation, giving way and antalgic gait	3 years	Positive ipsilateral SLR and impaired sensation power or reflexes	GP, physio, podiatry private chiropractor and specialist-physiotherapist	1	Nerve root block
**Catherine 60yrs Female**	Unemployed Shop worker	Leg pain, altered sensation and cramp; back ache (leg pain>back) and difficulty weight bearing	11.5 months	? Positive ipsilateral SLR (unclear data)	GP, physio, private physio and specialist-physiotherapist	2	Self-manage ment with review
**David 74yrs Male**	Retired professional	Leg pain>back pain and difficulty weight bearing	3 months	Impaired sensation power or reflexes	GP, physio, private physio and specialist-physiotherapist	1	Self-manage ment
**John 34yrs Male**	Unemployed delivery driver	Leg pain	9 months	Positive ipsilateral SLR	GP, physio, private physio, specialist-physiotherapist	3	Primary care physio
**Daniel 37yrs Male**	Shop worker: on sick leave	Leg and back pain; back spasm, leg giving way, saddle anaesthesia	21 months	Positive ipsilateral SLR and impaired sensation power or reflexes	GP and physio, specialist-physiotherapist and 2 nerve root blocks (at pain clinic)	2	Pain manage ment program me
**Janet 73yrs Female**	Retired	Leg pain back pain, leg giving way, foot numb, difficulty weight bearing	7 months	Positive ipsilateral SLR	GP, physio, private physio and specialist-physiotherapist	1	Nerve root block
**Bill 61yrs Male**	Part time manual worker	Leg pain, back pain, altered sensation in legs, leg giving way	10 months	Positive ipsilateral SLR and impaired sensation power or reflexes	GP and specialist-physiotherapist	1	**MDT review +

For peer review only

**Claire 45yrs Female**	Unemployed	Leg pain>back pain, altered sensation leg and foot	15 months	Impaired sensation power or reflexes	GP, physio and specialist-physiotherapist	2	Nerve root block
**Ruth 74yrs Female**	Retired shop worker	Leg pain	3 months	Positive ipsilateral SLR	GP, physio and specialist-physiotherapist	1	Physio (Unclear why nerve root block not offered)
**Henry 81yrs Male**	Retired manual worker	Leg pain and difficulty weight bearing	8 months	Positive ipsilateral SLR	GP, physio and specialist-physiotherapist	3	Review appt with spinal specialist
**Frances 72yrs Male**	Retired office worker	Leg pain and altered sensation in leg and foot	18 months – 2 years	Impaired sensation power or reflexes	GP, physio and spinal specialist. Specialist-physiotherapist and surgical opinion in last flare-up.	1	**MDT review
**Aisha 35yrs Female**	Office worker; currently off sick	Back and leg pain and altered sensation in legs	6 years (6 month this episode)	Positive ipsilateral SLR	GP, physio and spinal specialist	1	Surgical opinion
**Gareth 45 yrs Male**	Office and manual worker; on light duties	Leg>back pain and altered sensation and spasm in legs and feet	7 years	Positive ipsilateral SLR	GP, physio and specialist-physiotherapist	2	Offered but declined pain management
**Joanne 46yrs Female**	Housewife	Back and leg pain, altered sensation in legs and foot, saddle anaesthesia	3 years	Positive ipsilateral SLR	GP, physio and spinal specialist. Previous visit to spinal specialist. Pain management programme	2	Physio

* Magnetic resonance imaging (MRI) results categories: 1 Consistent with sciatica of nerve root origin. 2: Potentially relevant to symptoms but not consistent with sciatica of nerve root origin. 3: Do not appear relevant to patient's symptoms.
** Multidisciplinary team (MDT) review: meeting with pain clinic consultant and orthopaedic surgeon to ascertain

For peer review only - http://bmjopen.bmj.com/site/about/guidelines.xhtml

Our analysis explored how people experienced being managed for sciatica within an NHS care-pathway. We focused on the issues patients perceived mattered most and which had important implications for policy and practice. We present the findings under three thematic headings: (i) problems with the pathway (ii) required agency and (iii) agentic burden. We identify a series of problems with the pathway and show that they required patients to have agency (to be independent and proactive) to meet their healthcare needs. Agency was, however, difficult to enact and burdensome, and together with the pathway issues, negated patients' capability to manage sciatica. Patients' experience of the pathway is illustrated in Fig. 3.

Fig. 3 Patients' experience of the pathway

Fig. 4 Problems with the pathway

Theme 1 Problems with the pathway

Our data showed that a series of problems with the pathway (listed in Fig. 4) made it difficult for patients to access the management they perceived necessary. Participants reported that neither the way the pathway worked, nor the services available to them, including the risks, benefits and alternatives, were transparent.

> *He didn't tell me the procedure, I don't think he [GP] knows the procedure …you won't find it anywhere, it's only when you experience it that you know how it works. [Henry 81 years]*

Also, that it was difficult to contribute to management decisions because they were, by default, clinician-led or paternalistic.

> *I had no control over it [the next step], it was 'we're going to try this, we're going to do this.' [Daniel 37 years]*

Some participants reported that their specific needs and circumstances were not heard; some attributed this to care being protocol driven.

> *I just wanted to be heard, wanted them to actually listen … I feel like people with sciatica pains they are branded with the same stick...I'm sure the person next door with sciatica … would be given the exact kind of thing to do. [Aisha 35 years]*

> *She [physio] was just doing what she was told to do … she gave me five of the blueprint [exercise protocol] of what she had for my case. [Daniel 37 years]*

Accessing a specialist opinion (from either the specialist physiotherapist, or a surgical or pain clinic consultant) and investigations were reported to be difficult and protracted. This was because of the gatekeeping performed by their GP, physiotherapist and specialist physiotherapist, and having to first attend and fail physiotherapy.

> *I said 'Dr X [GP] suggested that actually it might be a good idea for you to refer me on to see a neuro-consultant … And he [spinal specialist] … said 'Let me just stop you there. … to be honest with you the neuro-consultant… they won't be interested in this at all'. [Gareth 45 years]*

Finally, pathway services appeared to be compartmentalised and non-collaborative. Apart from the multidisciplinary team meeting, where the specialist physiotherapist and consultant collaborated, services appeared to function with

For peer review only - http://bmjopen.bmj.com/site/about/guidelines.xhtml

limited co-operation, leaving patients to make sense of conflicting opinions and to join-up their own care. They were also required to join a new waiting list at each stage of the pathway, which resulted in recurrent delays.

> *She [GP] hasn't done a full [sickness] certificate because she said I have nothing on my screen to show the new results from the MRI… all this toing and froing adds to the stress. [Catherine, 60 years]*

> *They are sending me to the pain clinic, but I'm told there's an 18-week list… so I'm not looking at any quick fix. [Janet 73 years]*

Collectively these problems compromised the extent to which sciatica management was enabling; personalised; collaborative and involved patients; the result was a pathway that was insufficiently person-centred.

Theme 2 Required agency

Our data showed that to manage sciatica, patients were required to be independent and proactive. As well as being required to navigate the problems with the pathway, all participants reported that they independently sourced resources from outside of the NHS. They accessed factual information (through webpages or newspaper articles); the experiences of others who had experienced sciatica; and private treatment. Patients perceived these resources to be necessary to make sense of their management options, gain prompt access to treatment, or try options otherwise unavailable. Several participants also considered accessing surgery abroad, because it was quicker and easier to access than NHS surgery, and cheaper than private treatment in the UK.

> *I googled to try and find a direction in 'Well this is what's gone wrong, this is how you sort it out.' Or even a clue to say 'Fine, go to see your GP, try and point them in this direction'. [Gareth 45 years]*

> *[An acquaintance] went abroad because he felt like nothing was being done in this country soon enough…. [and] because it was cheaper [Aisha 35 years]*

Patients appeared to perceive that agency was required of a 'good' patient. This was illustrated in their desire to be seen as proactive, positive and compliant in their management.

> *I've looked into Pilates classes as well .. try and do anything that's going to ease it or help. [Claire, 45 years]*

> *The doctor suggest to me as well that I need to walk a lot. That's why I try my best you know to walk more. [John, 34 years]*

Theme 3 Agentic burden

Finally, being independent and proactive was difficult and burdensome. Participants reported that the impact of sciatica negated their ability to make decisions; to adhere to prescribed management; to navigate the pathway; and remain positive and motivated to self-manage.

> *It reduces me to tears and at times and I can't cope with that in my own mind somehow. I feel like I'm letting myself down by allowing it to take over. [Ruth, 74 years]*

> *I didn't want to feel a failure… I'm trying so hard to help myself, but it [exercise programme] was making it worse. [Joanne, 46 years]*

Participants also reported lacking the required skills to access and evaluate information. Furthermore, some were unaware of the need to critically appraise information and at times used information misaligned with the evidence to inform their management preferences.

For peer review only - http://bmjopen.bmj.com/site/about/guidelines.xhtml

I got a lot of the information from the internet, now I'm rubbish on the internet, somebody else got it for me…people who've had it, and what they've had done and it's got feedback and everything else. So all of this leads to the options that I just told you. [Henry 81 years]

Some also reported lacking the financial resources to access to private treatment, particularly when recurrent episodes of treatment, or costly interventions such as injections or surgery, were needed.

I don't have any money to go back there [abroad] for an operation [John, 34 years]

Finally, some participants lacked the support necessary for agency. Whilst some recruited others to help or act as their advocate, participants had varied access to such support.

I just went into physio, just did what I had to do. It was like I'd given up. [Catherine, 60 years]

DISCUSSION

To our knowledge, this paper is unique in exploring how patients experience the *process* of being managed within a care pathway. The findings are timely as NHS England implements its pathway; as other countries seek to understand the potential benefits(20); and because of the recent drive in health-policy for integrated, high-value care(7, 21). Although this is a small study, it is rich in detail and has conceptual transferability to similar services at an early stage of pathway implementation that manage patients who have not improved with conservative treatment and whose symptoms are sufficient to require investigation. Limitations include not undertaking iterative theoretical sampling during analysis and interviewing some participants before they had completed their management. These issues occurred as the methods were determined by the requirements of the original study. We identified three key themes about patients' experiences of being managed within a sciatica pathway: (i) problems with the pathway (ii) required agency and (iii) agentic burden.

We found that a series of problems with the sciatica pathway (insufficient transparency and information; clinician-led decisions; restricted access to specialist care; and a lack of collaboration between services) compromised patients' ability to access the management they perceived necessary. These findings concur with previous sciatica and low back pain studies(10, 13, 22-24) and are recognised in part in the national pathway as key issues to address. Our paper reveals that these issues remain even when management is provided within 'a pathway' and that patients perceive them to negate their ability to manage sciatica. We suggest that these problems are indicative of an insufficiently person-centred pathway resulting from a culture of paternalism, protocol-driven care, siloed services and cost-containment measures (designed to limit access to specialist care through requiring a protocol-driven, stepwise approach to management).

Aligning with previous studies(10, 13), we found that, to manage sciatica, patients independently accessed information, the experiences of others, and private healthcare. Aligning with Ellis et al's(23) study, which explored patients' perceptions of self-managing long-term condition, we found that participants perceived that agency (being independent and proactive) was necessary to be a 'good' patient. This paper furthers understanding by reframing patients' actions as required agency, necessary due to the problems with the pathway, and result in part due to the process of individualisation in healthcare that aims to reduce dependence on publicly provided healthcare, to introduce choice and competition; and to emphasise patients' rights and responsibilities(25).

For peer review only - http://bmjopen.bmj.com/site/about/guidelines.xhtml

Finally, our study found that required agency was difficult and burdensome to patients, because of the impact of sciatica and patients' lack of skill, funds and support, and alongside the problems with the pathway, this negated their ability to manage sciatica. Whilst new to the sciatica population, this finding aligns with the Burden of Treatment Theory (which recognises that when patients are inadequately supported by healthcare, this adversely affects their ability to manage their illness) (26), and is well-established in the literature exploring patients' experiences of managing long-term conditions(27). Our data emphasises the particular burden of required agency and the difficulty of enacting agency to manage sciatica without adequate support from healthcare.

Implications

Whilst it is now widely accepted that patient management should enable and support patient agency and be person-centred, this study reveals that, in this setting, these aspects of care were lacking. We therefore recommend that the sciatica-pathway and management options are made transparent to patients, and, align with the national pathway, emphasising the need for shared decision-making; information resources; collaborative services that enable rapid access to appropriate care; and for patients' experiences to be routinely explored as part of service evaluation. Finally, we highlight the need for management to empower and enable patients to manage sciatica with agency.

CONCLUSION

A series of problems with the pathway made it difficult for patients to access the management they perceived necessary. Patients were therefore required to have agency. Agency was, however, difficult to enact and burdensome whilst experiencing sciatica and because patients lacked the necessary skills, funds and support. Both the pathway issues and difficulty enacting agency negated patients' capability to manage sciatica. The key implication is the need for sciatica care-pathways to align with recommended best practice, and also to be more person-centred and to empower patient agency.

AUTHOR CONTRIBUTIONS: All authors contributed to the conception of this paper. CR and LR contributed to the conception and design of the study. CR performed data collection. CR, CP and LR contributed to material preparation and analysis. Each draft of the manuscript was written by CR and commented on by CP and LR. All authors read and approved the final manuscript.

DATA AVAILABILITY: The data sets generated during and/or analysed during the current study are not publicly available due to a restriction within the ethical agreement but are available from the corresponding author on reasonable request.

ACKNOWLEDGEMENTS: The authors gratefully acknowledge the support of study participants and the host NHS Trust.

COMPETING INTERESTS AND FUNDING: This research and preparation of the manuscript was funded by Health Education England (HEE) and the National Institute of Health Research (NIHR) as part of a Master's degree in Clinical Research and a Clinical Doctoral Research Fellowship (Round 4), awarded to CR, both undertaken at the University of Southampton. LR was funded, in part, by a NIHR Senior Clinical Lecturer award (Round 3). The NIHR and HEE had

For peer review only - http://bmjopen.bmj.com/site/about/guidelines.xhtml

no involvement in the preparation of the article for publication or the study design, data collection, analysis or write up of the study report.The authors have no other competing interests to declare.

For peer review only

For peer review only - http://bmjopen.bmj.com/site/about/guidelines.xhtml

REFERENCES

1. Albrecht DS, Ahmed SU, Kettner NW, et al. Neuroinflammation of the spinal cord and nerve roots in chronic radicular pain patients. Pain. 2018;159(5):968-77.
2. Koes BW, van Tulder MW, Peul WC. Diagnosis and treatment of sciatica. BMJ. 2007;334(7607):1313-7.
3. Konstantinou K, Dunn KM. Sciatica: review of epidemiological studies and prevalence estimates. Spine (Phila Pa 1976). 2008;33(22):2464-72.
4. Haugen AJ, Grovle L, Brox JI, et al. Estimates of success in patients with sciatica due to lumbar disc herniation depend upon outcome measure. Eur Spine J. 2011;20(10):1669-75.
5. Ryan C, Roberts L. 'Life on hold': The lived experience of radicular symptoms. A qualitative, interpretative inquiry. Musculoskelet Sci Pract. 2019;39:51-7.
6. Konstantinou K, Hider SL, Jordan JL, et al. The impact of low back-related leg pain on outcomes as compared with low back pain alone: a systematic review of the literature. Clin J Pain. 2013;29(7):644-54.
7. Curry N, Ham C. Clinical and Service Integration. The route to Improved Outcomes. London: The Kings Fund 2010.
8. National Institute of Health and Care Excellence (NICE). Low back pain and Sciatica in over 16s: Assessment and Management. 2016.
9. National Health Service (NHS) England. National Low back and radicular pain pathway. 2017.
10. Ong BN, Konstantinou K, Corbett M, et al. Patients' own accounts of sciatica: a qualitative study. Spine (Phila Pa 1976). 2011;36(15):1251-6.
11. Boote J, Newsome R, Reddington M, et al. Physiotherapy for Patients with Sciatica Awaiting Lumbar Micro-discectomy Surgery: A Nested, Qualitative Study of Patients' Views and Experiences. Physiother Res Int. 2017;22(3).
12. Ryan C, Roberts LC. Investigations for radiculopathy: The patient perspective. A qualitative, interpretative inquiry. Musculoskelet Sci Pract. 2018;33:71-6.
13. Goldsmith R, Williams NH, Wood F. Understanding sciatica: illness and treatment beliefs in a lumbar radicular pain population. A qualitative interview study. BJGP Open. 2019.
14. Baker SE, Edwards R. How many qualitative research intrviews is enough? ; 2014.
15. Guest. How many interviews are enough ? 2006.
16. Kongsted A, Kent P, Albert H, et al. Patients with low back pain differ from those who also have leg pain or signs of nerve root involvement - a cross-sectional study. BMC Musculoskelet Disord. 2012;13:236.
17. Bloor M. On the analysis of observational data: a discussion of the worth and uses of inductive techniques and participant validation. Sociology. 1978;12:545-52.
18. Braun V, Clarke V. Using thematic analysis in psychology. Qualitative Research in Psychology. 2006;3:77-101.
19. Ritchie J, Lewis J, Mcnaughton Nicholls C, et al Qualitative research practice : a guide for social science students and researchers. Second ed. London: Sage; 2014. p. 269-90.
20. Depreitere B, Jonckheer P, Desomer A, et al. The pivotal role for the multidisciplinary approach at all phases and at all levels in the national pathway for the management of low back pain and radicular pain in Belgium. Eur J Phys Rehabil Med. 2019.
21. Buchbinder R, van Tulder M, Oberg B, et al. Low back pain: a call for action. Lancet. 2018;391(10137):2384-8.
22.Hopayian K, Notley C. A systematic review of low back pain and sciatica patients' expectations and experiences of health care. Spine J. 2014;14(8):1769-80.

For peer review only

23. Ellis J, Boger E, Latter S, et al. Conceptualisation of the 'good' self-manager: A qualitative investigation of stakeholder views on the self-management of long-term health conditions. Soc Sci Med. 2017;176:25-33.
24. El-Haddad C, Damodaran A, Patrick McNeil H, et al. The experience of patients admitted to hospital with acute low back pain: a qualitative study. Int J Rheum Dis. 2018;21(4):796-803.
25. Galvin D. Disturbing notions of chronic illness and individual responsibility: towards a geneaology of morals. Health 2002;6:107-37.
26. May CR, Eton DT, Boehmer K, et al. Rethinking the patient: using Burden of Treatment Theory to understand the changing dynamics of illness. BMC Health Serv Res. 2014;14:281.
27. Demain S, Goncalves AC, Areia C, et al. Living with, managing and minimising treatment burden in long term conditions: a systematic review of qualitative research. PLoS One. 2015;10(5):e0125457.

For peer review only

For peer review only

For peer review only - http://bmjopen.bmj.com/site/about/guidelines.xhtml

Figures 1-4

Why managing sciatica is difficult: patients' experiences of a new national sciatica pathway. A qualitative, interpretative study.

Fig. 1

Leg pain and *at least one* of the following (on the affected leg): sensory deficit (to touch or pinprick); muscle weakness; impaired tendon reflex; a positive

Fig. 2

1. When and how did you first seek help for your leg pain?
   Probes: Who initiated, why, who did you see?

2. Who has treated you for leg pain?
   Probes: GP; Physio; physio specialist; others; how long with each; roles; coordination of care?

3. How were management decisions made?
   Probes: shared-decisions?

4. How did you move between the various clinicians?
   Probes: who initiated; shared-decision?

5. How have you managed your leg pain yourself?
   Probes: what helps, strategies tried

6. What aspects of your management have been most and least helpful?
   Probes: positive/ not so, what was most important/ useful

For peer review only

Fig. 3

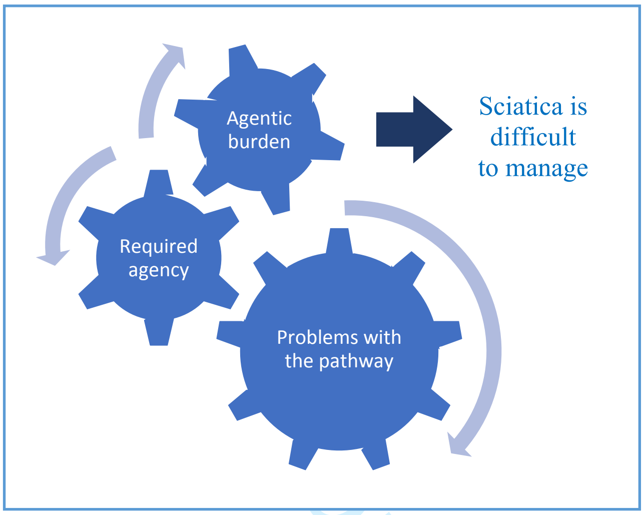

Fig. 4

- Insufficient transparency and information
- Clinician-led decisions
- Standardised care
- Restricted access to specialist care
- A lack of collaboration between services

For peer review only

Coding Tree

Super-ordinate themes	Themes	Categories	Codes
Why it is difficult for patients to manage sciatica	Problems with the pathway	Making sense of the pathway	Pathway transparency; Pathway information; Knowing the system
		Making sense of management options	Information about management options; Information about prognosis
		How decisions are made	Paternalism; Shared decision making; Protocol-driven care; Faith in the expert; Being grateful and not blaming; The treatment that's acceptable to me
		Standardised care failing to meet my needs	Lack of Individualised care; Unresponsive care; Patronising and dismissive attitudes; Being written off; Monitoring
		Reaching specialist care	An odd process; Required to have physio; Access to investigations; Seeking a specialist opinion; Reaching a diagnosis; Reaching the right person;
		Not moving forwards	Delays and waiting lists; Time to reach effective care; Not moving forwards
		Collaboration between services	Multiple clinicians; Compartmentalised care; Different professional opinions; Falling through the gaps; Lost in the system: MDT
	Required Agency	Navigating the pathway	Questioning expert opinion; Strategies to manage the consultation; Interweaving private and NHS care; Withdrawing from care
		Information seeking	Seeking the experiences of others; Self research; Making sense of my symptoms
		Helping myself	How can I help myself?; Making it work for me
		A good patient	I have tried; Maintaining a positive attitude; Being proactive; Being compliant
	Agentic burden	Why it is difficult to help myself	Helpless within the system; The impact of sciatica on agency; Required skills; Financial resources; Required support

Ryan et al **Why managing sciatica is difficult: patients' experiences of a new national sciatica pathway. A qualitative, interpretative study.**

For peer review only - http://bmjopen.bmj.com/site/about/guidelines.xhtml

Consolidated criteria for reporting qualitative studies (COREQ): 32-item checklist

No	Item	Guide questions/description	Alignment Page no.
**Domain 1: Research team and reflexivity**			
Personal Characteristics			
1.	Interviewer/facilitator	Which author/s conducted the interview or focus group?	4
2.	Credentials	What were the researcher's credentials? *E.g. PhD, MD*	4
3.	Occupation	What was their occupation at the time of the study?	4
4.	Gender	Was the researcher male or female?	4
5.	Experience and training	What experience or training did the researcher have?	4
Relationship with participants			

For peer review only

No	Item	Guide questions/description	Alignment Page no.
6.	Relationship established	Was a relationship established prior to study commencement?	4 (how participants were approached and participant exclusion criteria)
7.	Participant knowledge of the interviewer	What did the participants know about the researcher? e.g. *personal goals, reasons for doing the research*	4 (through the participant information sheet)
8.	Interviewer characteristics	What characteristics were reported about the interviewer/facilitator? e.g. *Bias, assumptions, reasons and interests in the research topic*	4 (researcher's occupation, sex, reason for undertaking the study)
**Domain 2: study design**			
Theoretical framework			
9.	Methodological orientation and Theory	What methodological orientation was stated to underpin the study? *e.g. grounded theory, discourse analysis, ethnography, phenomenology, content analysis*	3
Participant selection			

For peer review only

No	Item	Guide questions/description	Alignment Page no.
10.	Sampling	How were participants selected? *e.g. purposive, convenience, consecutive, snowball*	4
11.	Method of approach	How were participants approached? e.g. *face-to-face, telephone, mail, email*	4
12.	Sample size	How many participants were in the study?	5
13.	Non-participation	How many people refused to participate or dropped out? Reasons?	5
Setting			
14.	Setting of data collection	Where was the data collected? e.g. *home, clinic, workplace*	5
15.	Presence of non-participants	Was anyone else present besides the participants and researchers?	4
16.	Description of sample	What are the important characteristics of the sample? *e.g. demographic data, date*	5
Data collection			

For peer review only

No	Item	Guide questions/description	Alignment Page no.
17.	Interview guide	Were questions, prompts, guides provided by the authors? Was it pilot tested?	5
18.	Repeat interviews	Were repeat interviews carried out? If yes, how many?	5
19.	Audio/visual recording	Did the research use audio or visual recording to collect the data?	5
20.	Field notes	Were field notes made during and/or after the interview or focus group?	5
21.	Duration	What was the duration of the interviews or focus group?	5
22.	Data saturation	Was data saturation discussed?	4
23.	Transcripts returned	Were transcripts returned to participants for comment and/or correction?	5
**Domain 3: analysis and findings**			
Data analysis			
24.	Number of data coders	How many data coders coded the data?	5

For peer review only

No	Item	Guide questions/description	Alignment Page no.
25.	Description of the coding tree	Did authors provide a description of the coding tree?	Supplementary file S1
26.	Derivation of themes	Were themes identified in advance or derived from the data?	5
27.	Software	What software, if applicable, was used to manage the data?	5
28.	Participant checking	Did participants provide feedback on the findings?	5
Reporting			
29.	Quotations presented	Were participant quotations presented to illustrate the themes / findings? Was each quotation identified? e.g. *participant number*	8-10
30.	Data and findings consistent	Was there consistency between the data presented and the findings?	8-10
31.	Clarity of major themes	Were major themes clearly presented in the findings?	8-10
32.	Clarity of minor themes	Is there a description of diverse cases or discussion of minor themes?	8-10

For peer review only

BMJ Open

Why managing sciatica is difficult: patients' experiences of an NHS sciatica pathway. A qualitative interpretative study.

Journal:	*BMJ Open*
Manuscript ID	bmjopen-2020-037157.R1
Article Type:	Original research
Date Submitted by the Author:	25-Feb-2020
Complete List of Authors:	Ryan, Clare; Solent NHS Trust, Physiotherapy Service; University of Southampton, School of Health Sciences<br>Pope, Catherine; University of Oxford, Nuffield Department of Primary Care Health Sciences<br>Roberts, Lisa; University of Southampton, School of Health Sciences; University Hospital Southampton NHS Foundation Trust, Therapy Services Department
**Primary Subject Heading**:	Qualitative research
Secondary Subject Heading:	Patient-centred medicine, Rehabilitation medicine
Keywords:	Back pain < ORTHOPAEDIC & TRAUMA SURGERY, Musculoskeletal disorders < ORTHOPAEDIC & TRAUMA SURGERY, Adult orthopaedics < ORTHOPAEDIC & TRAUMA SURGERY, QUALITATIVE RESEARCH, Quality in health care < HEALTH SERVICES ADMINISTRATION & MANAGEMENT, Protocols & guidelines < HEALTH SERVICES ADMINISTRATION & MANAGEMENT

SCHOLARONE™
Manuscripts

I, the Submitting Author has the right to grant and does grant on behalf of all authors of the Work (as defined in the below author licence), an exclusive licence and/or a non-exclusive licence for contributions from authors who are: i) UK Crown employees; ii) where BMJ has agreed a CC-BY licence shall apply, and/or iii) in accordance with the terms applicable for US Federal Government officers or employees acting as part of their official duties; on a worldwide, perpetual, irrevocable, royalty-free basis to BMJ Publishing Group Ltd ("BMJ") its licensees and where the relevant Journal is co-owned by BMJ to the co-owners of the Journal, to publish the Work in this journal and any other BMJ products and to exploit all rights, as set out in our licence.

The Submitting Author accepts and understands that any supply made under these terms is made by BMJ to the Submitting Author unless you are acting as an employee on behalf of your employer or a postgraduate student of an affiliated institution which is paying any applicable article publishing charge ("APC") for Open Access articles. Where the Submitting Author wishes to make the Work available on an Open Access basis (and intends to pay the relevant APC), the terms of reuse of such Open Access shall be governed by a Creative Commons licence – details of these licences and which Creative Commons licence will apply to this Work are set out in our licence referred to above.

Other than as permitted in any relevant BMJ Author's Self Archiving Policies, I confirm this Work has not been accepted for publication elsewhere, is not being considered for publication elsewhere and does not duplicate material already published. I confirm all authors consent to publication of this Work and authorise the granting of this licence.

For peer review only

Clare Ryan[1&2] (ORCID ID: 0000-0002-3555-8624),

Professor Catherine Pope[3] (ORCID ID: 0000 0002 8935 6702)

Professor Lisa Roberts[2&4] (ORCHID ID: 0000-0003-2662-6696)

WHY MANAGING SCIATICA IS DIFFICULT: PATIENTS' EXPERIENCES OF AN NHS SCIATICA PATHWAY. A QUALITATIVE, INTERPRETATIVE STUDY.

[1]Physiotherapy Service, Solent NHS Trust, Portsmouth, UK.

[2]School of Health Sciences, University of Southampton, UK.

[3]Nuffield Department of Primary Care Health Sciences, University of Oxford, UK.

[4]Therapy Services Department, University Hospital Southampton NHS Foundation Trust, Southampton, UK.

Corresponding author: Clare Ryan, Physiotherapy Service, Solent NHS Trust, St Mary's Community Health Campus, Block A First Floor, Milton Road, Portsmouth, UK, PO3 6AD (Tel: +44 7531 332325). Email: Clare.ryan1@nhs.net

Word Count: 4,342

For peer review only

ABSTRACT

Objectives: Amid a political agenda for integrated, high-value care, the UK is implementing its Low Back and Radicular Pain Pathway. To align care with need, it is imperative to understand the patients' perspective. The purpose of this study was therefore, to explore how people experience being managed for sciatica within an NHS pathway.

Design: Qualitative interpretative study.

Setting: Musculoskeletal Service in an NHS, Primary Care Trust, UK.

Participants: The sample comprised 14 people aged ≥18years with a clinical presentation of sciatica, who were currently under the care of a specialist physiotherapist (the specialist spinal triage practitioner), had undergone investigations (magnetic resonance imaging) and received the results within the past six weeks. People were excluded if they had previously undergone spinal surgery or if the suspected cause of symptoms was cauda equina syndrome or sinister pathology. Participants were sampled purposively for variation in age and gender. Data were collected using individual semi-structured interviews (duration: 38-117 minutes; median: 82.6 minutes), which were audio-recorded and transcribed verbatim. Data were analysed thematically.

Results: A series of problems with the local pathway (insufficient transparency and information; clinician-led decisions; standardised management; restricted access to specialist care; and a lack of collaboration between services) made it difficult for patients to access the management they perceived necessary. Patients were therefore required to be independent and proactive or have agency. This was, however, difficult to achieve (due to the impact of sciatica and because patients lacked the necessary skills, funds and support) and together with the pathway issues, this negated patients' capability to manage sciatica.

Conclusions: This novel paper explores how patients experience the process of being managed within a sciatica pathway. Whilst highlighting the need to align with recommended best practice, it shows the need to be more person-centred and to support and empower patient agency.

Keywords: Agency; Pathway; Patient experience; Qualitative Research; Sciatica; Treatment Burden.

ClinicalTrials.gov reference: UOS-2307-CR

ARTICLE SUMMARY

Strengths and limitations of this study

- To our knowledge, this paper is unique in exploring how patients with sciatica experience the *process* of being managed within a pathway.
- Although this is a small, single site study it is rich in detail and has conceptual transferability to similar services at an early stage of pathway implementation managing patients who have not improved with conservative treatment and whose symptoms are sufficient to require investigation.
- Limitations include not undertaking iterative theoretical sampling during analysis and interviewing some participants before they had completed their management.

For peer review only

For peer review only - http://bmjopen.bmj.com/site/about/guidelines.xhtml

INTRODUCTION

Sciatica is characterised by pain, altered sensation and/or weakness in the leg. It is thought to result from compression, inflammation and/or sensitisation of a lumbar/sacral nerve root, caused by lumbar disc-herniation and stenosis, or rarely, sinister pathology (such as cancer); however, it is not always possible to identify a structural cause(1, 2). Sciatica differs from somatic referred leg pain which is attributed to structures other than the nerve root such as the joints, muscles and ligaments. It is well established that the presence of sciatica, compared to low back pain alone, or somatic referred leg pain, increases symptom severity, disability, absence from work and negatively impacts on health outcome(3). Furthermore, patients report that sciatica can be incapacitating and severely limit daily activities(4). Sciatica prevalence estimates vary widely, from 1.2% to 43%, reflecting differing diagnostic criteria and sampling methods(5). Whilst the majority of patients with sciatica experience early improvement in symptoms, usually in the first 2-3 months, either with or without treatment; a minority will experience more persistent symptoms or disability, and for some this continues beyond 12 months. Some patients will experience intermittent or recurrent sciatic symptoms over time. There is, however, inconsistency in the literature about the proportion of patients affected by ongoing symptoms. Whilst one review found this to be as few as 13% of patients at 6 months(6), another study based in primary care, found that for 45% of patients, disability had failed to significantly improve at 12 months(7). The reason for this variation is not fully understood but may be associated with symptom duration at presentation or the type of studies to which patients were recruited (clinical versus epidemiological). Recent work indicates that factors negatively associated with recovery include longer leg pain duration; more symptoms associated with sciatica; and patient's belief that the problem would last a long time; conversely, having myotomal weakness was positively associated with recovery(7).

Over the past decade, in the UK and the developed world, there has been a political agenda for healthcare that is integrated (collaborative and responsive) and high-value (aligns with best practice)(8). Reflecting these agendas, and to facilitate implementation of recent National Institute of Health and Care Excellence (NICE) guidance(9), a UK Low Back and Radicular Pain Pathway (10) has been developed. Achieving integrated, high-value care is particularly important for patients with sciatica who commonly cross sectors of care to access management from a range of health-professionals. The new national pathway recommends that patients with sciatica are initially managed within primary care, with medication and/or physiotherapy, with a 2-week review after initial presentation. For those with severe pain that fails to improve, assessment by a specialist (spinal triage) practitioner is recommended at 2-6 weeks, or at 6 weeks for those with non-tolerable pain. In the UK this specialist role is commonly performed by an advanced practice physiotherapist(10). The pathway recommends that investigations such as magnetic resonance imaging (MRI) are offered (if they are likely to change management) and completed within 4 weeks, with results review within 2 weeks. Where imaging supports the suspected clinical diagnosis of sciatica, referral for an epidural injection or surgery is advised, with surgery occurring within 8-12 weeks of the onset of symptoms, or for those with very severe symptoms, within 3 weeks. When no concordant structural cause is identified, or the patient wishes to avoid invasive intervention, the recommended approach is a combined physical and psychological programme. The pathway advocates that clinicians share decision making with patients at each stage of the pathway.

Understanding patients' experiences of being managed for sciatica can inform the provision of care. Four qualitative UK based studies(11-14) with a total of 75 patients have provided some insight into patients' beliefs, expectations, and experiences of components of the conservative management of sciatica. These papers do not, however, provide an

For peer review only - http://bmjopen.bmj.com/site/about/guidelines.xhtml

overview of what it is like to be managed in a sciatica pathway. This paper therefore addresses the question, 'what are patients' experiences of being managed within an NHS sciatica pathway?'

METHODS

The data for this paper were collected as part of a wider study about patients' experiences of investigations for sciatica(13). Ethical approval was gained from the South-West Ethics Committee in September 2015 (15/SW/0247). This paper is reported in line with the COREQ guidelines.

Study design

This was an interpretative, qualitative research study, a methodology consistent with the aim of exploring peoples' experiences.

Setting

The setting was an outpatient physiotherapy service of a UK, NHS, Community Trust. This service was selected as it was working to align its practice with the new national pathway and had experienced specialist spinal triage practitioners. At the time data were collected, the first version of the national pathway had been available for 16 months. Over this period, changes had occurred in the spinal surgery, injection and imaging providers. Not all providers were commissioned and funded by the same organisation. Issues with staff shortages and provider negotiations had resulted in waiting times of 6 months for a routine appointment with a spinal surgeon and 4 months for an injection (however, patients could choose to access this in the adjacent city within 4 weeks). Following a recent change in provider, the wait for imaging had reduced from 6 to 3 weeks.

Participants and recruitment

The sample comprised people aged ≥18years with a clinical presentation of sciatica, who were currently under the care of a specialist-physiotherapist (the specialist-spinal-triage-practitioner), had undergone investigations (magnetic resonance imaging (MRI)) and received the results within the past six weeks. Participants were therefore recruited at a key point in their pathway management, when they had failed initial conservative management, had undergone investigations to determine whether a concordant structural cause could be identified, and had discussed with a specialist-physiotherapist the most appropriate next step.

The study was designed to recruit 12-15 participants. No claims were made to reach data saturation, as each person's experience of a phenomenon is unique. However, aligning with qualitative research recommendations(15, 16), this number was considered enough to enable a rich, detailed analysis of this defined, relatively homogenous group, whilst providing sufficient information to answer the research question, and sufficient variation within the sample to enhance transferability.

The clinical diagnosis of sciatica was made by the patient's specialist physiotherapist using information from their clinical assessment and based on diagnostic criteria (Fig. 1) reflecting those used in practice(17). The patient's radiological findings did not inform this diagnosis. The specialist physiotherapists were clinicians with ≥ 10 years musculoskeletal experience, who undertook specialist spinal training ≥ four times a year. We used purposive sampling

For peer review only - http://bmjopen.bmj.com/site/about/guidelines.xhtml

For peer review only

to gain representation across age, sex and duration of symptoms, and recruited 14 participants, sufficient to enable rich, detailed analysis.

Fig. 1 Criteria for diagnosing sciatica(17)

Potential participants were approached about participating in the study, by their specialist physiotherapist, when they attended for their investigation results. Those interested were provided with verbal and written information about the study. With their written consent, the researcher made contact to arrange an interview date. To increase the homogeneity of the sample, people were excluded if: they had previously undergone spinal surgery or the suspected cause of symptoms was sinister pathology or cauda equina syndrome. Patients were also excluded if they were unable to communicate without the assistance of an interpreter; they lacked capacity to provide consent; or the researcher had treated them in a previous episode of back pain (to minimise bias).

Data collection

The lead author (CR) a female specialist physiotherapist and MRes student (with prior experience and training in conducting qualitative interviews) collected data between October 2015 and May 2016 using in-depth, individual, face-to-face, semi-structured interviews. This method was selected to facilitate rapport and enable emergent issues to be explored. To minimise the influence of the researcher's position, CR introduced herself as a researcher, and hosted the interviews in an individual room within the hospital, away from the physiotherapy department.

A topic guide was used as a starting point for discussion, however, interviews followed the participant's lead, with emergent issues being explored and, where indicated, incorporated into subsequent interviews. Fig. 2 details the key questions used to explore patients' experience of the pathway. Minimal facilitation was used to prevent 'leading'. At the start of data collection, CR conducted pilot interviews with two participants to provide face and content validity for the questions asked. As the content and key wording of questions were substantially unchanged, the data from these participants were included in the study. The interviews were audio-recorded, transcribed verbatim, and pseudonyms were used to maintain anonymity. We did not ask participants to validate the transcripts or findings as the usefulness of this strategy is contested(18).

Fig. 2 Topic guide

Patient and public involvement

Informal discussions with a series of patients, and engagement with the public during 3 dissemination events, aided the interpretation of findings.

Data analysis

We analysed the data manually, thematically and iteratively, based on the method of Braun and Clarke(19). Thematic analysis has three stages: (i) line-by-line coding, (ii) developing descriptive themes and (iii) generating analytical themes. Firstly, we explored the data on its own terms, using inductive line-by-line coding. The data relevant to the research question were identified, explored through selective and axial coding, and initial concepts identified. We then

For peer review only - http://bmjopen.bmj.com/site/about/guidelines.xhtml

explored relationships and potential explanations for concepts, moving back and forth between the raw data, key concepts and relevant clinical, theoretical and policy literature. We used charts to manage the data, to facilitate comparison within and between cases, and to ensure that analysis remained rooted in the data(20). We included variation and complexity and used analytical and reflexive memos to facilitate a deeper understanding(20). Each of the authors contributed to analysis; CR identified the initial codes and concepts, which CP and LR, expert qualitative researchers, interrogated to refine their scope; relevance; constituent parts; relationships; and explanatory potential. Involving three analysts with different professional backgrounds aided the rigor of analysis and provided a check to minimise potential bias, assumptions and data selection.

RESULTS

The sample comprised fourteen participants; aged 34-81 years (median 61 years); eight participants were female. Participants described a three-month to nine-year (median thirteen months) duration of symptoms. By the time of their interview, half of participants had experienced improvement in their symptoms, including 5 with significant improvement. Participant characteristics are detailed in Table 1. Four additional people were identified but not included as they did not meet inclusion criteria (n=1); did not wish to participate (n=1); attended on the wrong day (n=1); or could not be contacted (n=1). Interviews ranged in length from 38-117 minutes (median 82.6 minutes).

Our analysis explored how people experienced being managed for sciatica within an NHS pathway. We focused on the issues patients perceived mattered most and which had important implications for policy and practice. We present the findings under three thematic headings: (i) problems with the pathway (ii) required agency and (iii) the burden of agency. The term 'agency' refers to the independent and proactive actions undertaken by a patient as part of the self-management of their health(21). We identify a series of problems with the pathway and show that they required patients to be independent and proactive, or have agency, to meet their healthcare needs. Being independent and proactive was, however, difficult and burdensome for patients to achieve, and together with the pathway issues, negated patients' capability to manage sciatica. Patients' experience of the pathway is illustrated in Fig. 3.

Fig. 3 Patients' experience of the pathway

Fig. 4 Problems with the pathway

Theme 1 Problems with the pathway

Our data showed that a series of problems with the pathway (listed in Fig. 4) made it difficult for patients to access the management they perceived necessary. Participants reported that neither the way the pathway worked, nor the services available to them, including the risks, benefits and alternatives, were transparent.

> *He didn't tell me the procedure, I don't think he [GP] knows the procedure ...you won't find it anywhere, it's only when you experience it that you know how it works. [Henry 81 years]*

For peer review only - http://bmjopen.bmj.com/site/about/guidelines.xhtml

Table 1 Participant characteristics(4, 13)

Participant age and sex	Work status and occupation	Symptoms	Symptom duration	Neurological findings	Pathway management for this episode of sciatica	MRI findings*	Likely next step
**Claire 45yrs Female**	Unemployed	Leg pain>back pain, altered sensation leg and foot	15 months	Impaired sensation power or reflexes	GP, physio and specialist-physiotherapist	2	Nerve root block
**Julia 63yrs Female**	Part time office worker	Leg pain, altered sensation, giving way and antalgic gait	3 years	Positive ipsilateral SLR and impaired sensation power or reflexes	GP, physio, podiatry private chiropractor and specialist-physiotherapist	1	Nerve root block
**Catherine 60yrs Female**	Unemployed Shop worker	Leg pain, altered sensation and cramp; back ache (leg pain>back) and difficulty weight bearing	11.5 months	? Positive ipsilateral SLR (unclear data)	GP, physio, private physio and specialist-physiotherapist	2	Self-management with review
**David 74yrs Male**	Retired professional	Leg pain>back pain and difficulty weight bearing	3 months	Impaired sensation power or reflexes	GP, physio, private physio and specialist-physiotherapist	1	Self-management
**John 34yrs Male**	Unemployed delivery driver	Leg pain	9 months	Positive ipsilateral SLR	GP, physio, private physio, specialist-physiotherapist	3	Primary care physio
**Daniel 37yrs Male**	Shop worker: on sick leave	Leg and back pain; back spasm, leg giving way, saddle anaesthesia	21 months	Positive ipsilateral SLR and impaired sensation power or reflexes	GP and physio, specialist-physiotherapist and 2 nerve root blocks (at pain clinic)	2	Pain management programme
**Janet 73yrs Female**	Retired	Leg pain back pain, leg giving way, foot numb, difficulty weight bearing	7 months	Positive ipsilateral SLR	GP, physio, private physio and specialist-physiotherapist	1	Nerve root block
**Bill 61yrs Male**	Part time manual worker	Leg pain, back pain, altered sensation in legs, leg giving way	10 months	Positive ipsilateral SLR and impaired sensation power or reflexes	GP and specialist-physiotherapist	1	**MDT review +

For peer review only

**Ruth 74yrs Female**	Retired shop worker	Leg pain	3 months	Positive ipsilateral SLR	GP, physio and specialist-physiotherapist	1	Physio (Unclear why nerve root block not offered)
**Henry 81yrs Male**	Retired manual worker	Leg pain and difficulty weight bearing	8 months	Positive ipsilateral SLR	GP, physio and specialist-physiotherapist	3	Review appt with spinal specialist
**Frances 72yrs Male**	Retired office worker	Leg pain and altered sensation in leg and foot	18 months – 2 years	Impaired sensation power or reflexes	GP, physio and spinal specialist. Specialist-physiotherapist and surgical opinion in last flare-up.	1	**MDT review
**Aisha 35yrs Female**	Office worker; currently off sick	Back and leg pain and altered sensation in legs	6 years (6 month this episode)	Positive ipsilateral SLR	GP, physio and spinal specialist	1	Surgical opinion
**Gareth 45 yrs Male**	Office and manual worker; on light duties	Leg>back pain and altered sensation and spasm in legs and feet	7 years	Positive ipsilateral SLR	GP, physio and specialist-physiotherapist	2	Offered but declined pain management
**Joanne 46yrs Female**	Housewife	Back and leg pain, altered sensation in legs and foot, saddle anaesthesia	3 years	Positive ipsilateral SLR	GP, physio and spinal specialist. Previous visit to spinal specialist. Pain management programme	2	Physio

* Magnetic resonance imaging (MRI) results categories: 1 Consistent with sciatica of nerve root origin. 2: Potentially relevant to symptoms but not consistent with sciatica of nerve root origin. 3: Do not appear relevant to patient's symptoms.
** Multidisciplinary team (MDT) review: meeting with pain clinic consultant and orthopaedic surgeon to ascertain

For peer review only

Also, it was difficult for patients to contribute to management decisions because they were, by default, clinician-led or paternalistic.

> *I had no control over it [the next step], it was 'we're going to try this, we're going to do this.' [Daniel 37 years]*

Some participants reported that their specific needs and circumstances were not heard; some attributed this to care being protocol driven.

> *I just wanted to be heard, wanted them to actually listen … I feel like people with sciatica pains they are branded with the same stick...I'm sure the person next door with sciatica … would be given the exact kind of thing to do. [Aisha 35 years]*

> *She [physio] was just doing what she was told to do … she gave me five of the blueprint [exercise protocol] of what she had for my case. [Daniel 37 years]*

Accessing a specialist opinion (from either the specialist physiotherapist, or a surgical or pain clinic consultant) and investigations were reported to be difficult and protracted. This was because of the gatekeeping performed by their GP, physiotherapist and specialist physiotherapist, and having to first attend and fail physiotherapy.

> *I said 'Dr X [GP] suggested that actually it might be a good idea for you to refer me on to see a neuro-consultant ... And he [spinal specialist] ... said 'Let me just stop you there. ... to be honest with you the neuro-consultant… they won't be interested in this at all'. [Gareth 45 years]*

Finally, pathway services appeared to be compartmentalised and non-collaborative. Apart from the multidisciplinary team meeting, where the specialist physiotherapist and consultant collaborated, services appeared to function with limited co-operation, leaving patients to make sense of conflicting opinions and to join-up their own care. They were also required to join a new waiting list at each stage of the pathway, which resulted in recurrent delays.

> *She [GP] hasn't done a full [sickness] certificate because she said I have nothing on my screen to show the new results from the MRI… all this toing and froing adds to the stress. [Catherine, 60 years]*

> *They are sending me to the pain clinic, but I'm told there's an 18-week list… so I'm not looking at any quick fix. [Janet 73 years]*

Collectively these problems compromised the extent to which sciatica management was enabling; personalised; collaborative and involved patients; the result was a pathway that was insufficiently person-centred.

Theme 2 Required agency

Our data showed that to manage sciatica, patients were required to be independent and proactive. As well as being required to navigate the problems with the pathway, all participants reported that they independently sourced resources from outside of the NHS. They accessed factual information (through webpages or newspaper articles); the experiences of others who had experienced sciatica; and private treatment. Patients perceived these resources to be necessary to make sense of their management options, gain prompt access to treatment, or try options otherwise unavailable. Several participants also considered accessing surgery abroad, because it was quicker and easier to access than NHS surgery, and cheaper than private treatment in the UK.

> *I googled to try and find a direction in 'Well this is what's gone wrong, this is how you sort it out.' Or even a clue to say 'Fine, go to see your GP, try and point them in this direction'. [Gareth 45 years]*

> *[An acquaintance] went abroad because he felt like nothing was being done in this country soon enough…. [and] because it was cheaper [Aisha 35 years]*

For peer review only - http://bmjopen.bmj.com/site/about/guidelines.xhtml

Patients perceived that agency was required of a 'good' patient, illustrated in their accounts of being proactive, positive and compliant.

> *I've looked into Pilates classes as well ... try and do anything that's going to ease it or help. [Claire, 45 years]*

> *The doctor suggest to me as well that I need to walk a lot. That's why I try my best you know to walk more. [John, 34 years]*

Theme 3 The burden of agency

Finally, being independent and proactive was difficult and burdensome. Participants reported that the impact of sciatica negated their ability to make decisions; to adhere to prescribed management; to navigate the pathway; and remain positive and motivated to self-manage.

> *It reduces me to tears and at times and I can't cope with that in my own mind somehow. I feel like I'm letting myself down by allowing it to take over. [Ruth, 74 years]*

> *I didn't want to feel a failure… I'm trying so hard to help myself, but it [exercise programme] was making it worse. [Joanne, 46 years]*

Participants also reported lacking the required skills to access and evaluate information. Some were unaware of the need to critically appraise information and at times used information misaligned with the evidence to inform their management preferences.

> *I got a lot of the information from the internet, now I'm rubbish on the internet, somebody else got it for me…people who've had it, and what they've had done, and it's got feedback and everything else. So, all of this leads to the options that I just told you. [Henry 81 years]*

Some reported lacking the financial resources to access to private treatment, especially if multiple treatments, or costly interventions such as injections or surgery, were needed.

> *I don't have any money to go back there [abroad] for an operation [John, 34 years]*

Finally, some participants lacked the support necessary for agency.

> *I just went into physio, just did what I had to do. It was like I'd given up. [Catherine, 60 years]*

DISCUSSION

To our knowledge, this paper is unique in exploring how patients experience the *process* of being managed within a sciatica pathway. The findings are timely as NHS England implements its pathway and as other countries seek to understand the potential benefits(22); and because of the recent drive in health-policy for integrated, high-value care(8, 23). Although this is a small study, it is rich in detail and has conceptual transferability to similar services that manage patients who have not improved with conservative treatment and whose symptoms are sufficient to require investigation. Limitations include not undertaking iterative theoretical sampling during analysis (because analysis for this paper was completed after data collection had ended) and interviewing some participants before they had completed their management (to align with the aim of the original study, patients were interviewed within 6 weeks of undergoing investigations).

We identified three key themes about patients' experiences of being managed within a sciatica pathway: (i) problems with the pathway (ii) required agency and (iii) the burden of agency. We found that problems with the local sciatica

For peer review only - http://bmjopen.bmj.com/site/about/guidelines.xhtml
For peer review only

pathway compromised patients' ability to access the management they perceived necessary. These included insufficient transparency and information; clinician-led decisions; restricted access to specialist care; and a lack of collaboration between services. These issues have previously been noted in the sciatica and low back pain literature(11, 14, 24-26). They are also already recognised in the national pathway as important issues to address. We suggest that because the local pathway focused on delivering the 'mechanics' of the pathway (procuring services; enabling faster access to imaging; and setting up regular case discussion meetings) it did not prioritise ensuring that management was person-centred. Our data reveals a culture of paternalism, protocol-driven care, information being provided on a 'need to know basis', and siloed services. Patients' frustration with the lack of timely access to a specialist opinion, likely reflects in part, the known issues with staff shortages and difficulty negotiating provider contracts. However, in addition, the national pathway places a new emphasis on enabling rapid access to imaging and injection or surgery (for those with severe or intolerable symptoms and a relevant cause identified on imaging) and achieving this requires a shift in practice and resources. Whilst this approach reflects expert opinion, it is in contrast to the established 'stepped model' of sciatica care that advocates the more limited use of invasive interventions, with evidenced cost effectiveness(27). We suggest that until the national pathway is underpinned by evidence of its clinical and cost effectiveness, services may be ambivalent about making the changes necessary to align with its timelines. Whilst these issues are specific to the local pathway, they seem likely to apply to other services in the early stages of pathway implementation.

Aligning with previous studies(11, 14), we found that, to manage sciatica, patients independently accessed information; the experiences of others; and private healthcare. They also perceived that a 'good' patient was necessarily independent and proactive. Whilst new to the sciatica literature, these findings align with the experiences of those self-managing long term conditions(25). This paper furthers understanding by reframing patients' actions as agency, and arguing that this agency becomes necessary in part due to the problems with the pathway as well as in response to wider neo-liberal expectations that people manage their own health and reduce dependence on the state (28). This study shows that it has become normal for both patients and staff to expect patients to be proactive and independent in their management. However, our study also found that the requirement to be independent and proactive when managing sciatica was difficult and burdensome to patients. This was because of the impact of sciatica and patients' lack of skill, funds and support. The Burden of Treatment Theory recognises that when patients are expected to participate in their healthcare but lack adequate support, this adversely affects their ability to manage their illness (29). Our analysis demonstrates the particular burden of being required to navigate the pathway issues and to independently and proactively manage sciatica without the necessary support.

Implications

Local implications include the need to improve and then re-evaluate the following aspects of care: patient information about the pathway; staff training and patient resources to enable effective shared decision making; collaborative, joined up working; and timely progression through the pathway. Also, patients' experiences should be routinely explored as part of service evaluation. Transferable implications include the need to ensure pathway care is person-centred and provides the support and resources necessary to enable and empower patients to manage sciatica independently and proactively.

For peer review only - http://bmjopen.bmj.com/site/about/guidelines.xhtml

Further research exploring patients' experiences of the national pathway, particularly in services in which the new pathway is fully implemented, would enable a more comprehensive understanding of patients' experience of being managed in NHS sciatica pathways.

CONCLUSION

A series of problems with the local pathway made it difficult for patients to access the management they perceived necessary. Patients were required to be independent and proactive in their management, but this was difficult to achieve and burdensome, due to sciatica symptoms, and lack of skills, funds and support. Both the pathway issues and the requirement to be independent and proactive negated patients' capability to manage sciatica. The key implications are the need for sciatica pathways to align with recommended best practice, and also to be more person-centred and to empower and enable patients to manage sciatica independently and proactively.

AUTHOR CONTRIBUTIONS: All authors contributed to the conception of this paper. CR and LR contributed to the conception and design of the study. CR performed data collection. CR, CP and LR contributed to material preparation and analysis. Each draft of the manuscript was written by CR and commented on by CP and LR. All authors read and approved the final manuscript.

DATA AVAILABILITY: The data sets generated during and/or analysed during the current study are not publicly available due to a restriction within the ethical agreement but are available from the corresponding author on reasonable request.

ACKNOWLEDGEMENTS: The authors gratefully acknowledge the support of study participants and the host NHS Trust.

COMPETING INTERESTS AND FUNDING: This research and preparation of the manuscript was funded by Health Education England (HEE) and the National Institute of Health Research (NIHR) as part of a Master's degree in Clinical Research and a Clinical Doctoral Research Fellowship (Round 4), awarded to CR, both undertaken at the University of Southampton. LR was funded, in part, by a NIHR Senior Clinical Lecturer award (Round 3). The NIHR and HEE had no involvement in the preparation of the article for publication or the study design, data collection, analysis or write up of the study report. The authors have no other competing interests to declare.

REFERENCES

1. Albrecht DS, Ahmed SU, Kettner NW, Borra RJH, Cohen-Adad J, Deng H, et al. Neuroinflammation of the spinal cord and nerve roots in chronic radicular pain patients. Pain. 2018;159(5):968-77.
2. Koes BW, van Tulder MW, Peul WC. Diagnosis and treatment of sciatica. BMJ. 2007;334(7607):1313-7.
3. Konstantinou K, Hider SL, Jordan JL, Lewis M, Dunn KM, Hay EM. The impact of low back-related leg pain on outcomes as compared with low back pain alone: a systematic review of the literature. Clin J Pain. 2013;29(7):644-54.
4. Ryan C, Roberts L. 'Life on hold': The lived experience of radicular symptoms. A qualitative, interpretative inquiry. Musculoskelet Sci Pract. 2019;39:51-7.

For peer review only - http://bmjopen.bmj.com/site/about/guidelines.xhtml

5. Konstantinou K, Dunn KM. Sciatica: review of epidemiological studies and prevalence estimates. Spine (Phila Pa 1976). 2008;33(22):2464-72.
6. Alentado VJ, Lubelski D, Steinmetz MP, Benzel EC, Mroz TE. Optimal duration of conservative management prior to surgery for cervical and lumbar radiculopathy: a literature review. Global Spine J. 2014;4(4):279-86.
7. Konstantinou K, Dunn KM, Ogollah R, Lewis M, van der Windt D, Hay EM, et al. Prognosis of sciatica and back-related leg pain in primary care: the ATLAS cohort. Spine J. 2018;18(6):1030-40.
8. Curry N, Ham C. Clinical and Service Integration. The route to Improved Outcomes. London: The Kings Fund 2010.
9. National Institute of Health and Care Excellence (NICE). Low back pain and Sciatica in over 16s: Assessment and Management. 2016.
10. National Health Service (NHS) England. National Low back and radicular pain pathway. 2017.
11. Ong BN, Konstantinou K, Corbett M, Hay E. Patients' own accounts of sciatica: a qualitative study. Spine (Phila Pa 1976). 2011;36(15):1251-6.
12. Boote J, Newsome R, Reddington M, Cole A, Dimairo M. Physiotherapy for Patients with Sciatica Awaiting Lumbar Micro-discectomy Surgery: A Nested, Qualitative Study of Patients' Views and Experiences. Physiother Res Int. 2017;22(3).
13. Ryan C, Roberts LC. Investigations for radiculopathy: The patient perspective. A qualitative, interpretative inquiry. Musculoskelet Sci Pract. 2018;33:71-6.
14. Goldsmith R, Williams NH, Wood F. Understanding sciatica: illness and treatment beliefs in a lumbar radicular pain population. A qualitative interview study. BJGP Open. 2019.
15. Baker SE, Edwards R. How many qualitative research intrviews is enough? ; 2014.
16. Guest. How many interviews are enough ? 2006.
17. Kongsted A, Kent P, Albert H, Jensen TS, Manniche C. Patients with low back pain differ from those who also have leg pain or signs of nerve root involvement - a cross-sectional study. BMC Musculoskelet Disord. 2012;13:236.
18. Bloor M. On the analysis of observational data: a discussion of the worth and uses of inductive techniques and participant validation. Sociology. 1978;12:545-52.
19. Braun V, Clarke V. Using thematic analysis in psychology. Qualitative Research in Psychology. 2006;3:77-101.
20. Ritchie J, Lewis J, Mcnaughton Nicholls C, Ormston R. Qualitative research practice : a guide for social science students and researchers. Second ed. London: Sage; 2014. p. 269-90.
21. Armstrong D. Actors, patients and agency: a recent history. Sociology of Health and Illness. 2014;36(2):163-74.
22. Depreitere B, Jonckheer P, Desomer A, Van Wambeke P. The pivotal role for the multidisciplinary approach at all phases and at all levels in the national pathway for the management of low back pain and radicular pain in Belgium. Eur J Phys Rehabil Med. 2019.
23. Buchbinder R, van Tulder M, Oberg B, Costa LM, Woolf A, Schoene M, et al. Low back pain: a call for action. Lancet. 2018;391(10137):2384-8.
24. Hopayian K, Notley C. A systematic review of low back pain and sciatica patients' expectations and experiences of health care. Spine J. 2014;14(8):1769-80.
25. Ellis J, Boger E, Latter S, Kennedy A, Jones F, Foster C, et al. Conceptualisation of the 'good' self-manager: A qualitative investigation of stakeholder views on the self-management of long-term health conditions. Soc Sci Med. 2017;176:25-33.
26. El-Haddad C, Damodaran A, Patrick McNeil H, Hu W. The experience of patients admitted to hospital with acute low back pain: a qualitative study. Int J Rheum Dis. 2018;21(4):796-803.

For peer review only

27. Fitzsimmons D, Phillips CJ, Bennett H, Jones M, Williams N, Lewis R, et al. Cost-effectiveness of different strategies to manage patients with sciatica. Pain. 2014;155(7):1318-27.
28. Galvin D. Disturbing notions of chronic illness and individual responsibility: towards a geneaology of morals. Health 2002;6:107-37.
29. May CR, Eton DT, Boehmer K, Gallacher K, Hunt K, MacDonald S, et al. Rethinking the patient: using Burden of Treatment Theory to understand the changing dynamics of illness. BMC Health Serv Res. 2014;14:281.

For peer review only

For peer review only

For peer review only - http://bmjopen.bmj.com/site/about/guidelines.xhtml

Fig. 1

Leg pain and *at least one* of the following (on the affected leg): sensory deficit (to touch or pinprick); muscle weakness; impaired tendon reflex; a positive straight leg raise reproducing the person's familiar pain; or a positive prone knee bend test combined with anterior thigh pain.

For peer review only

Fig. 2

1. When and how did you first seek help for your leg pain?
   Probes: Who initiated, why, who did you see?

2. Who has treated you for leg pain?
   Probes: GP; Physio; physio specialist; others; how long with each; roles; coordination of care?

3. How were management decisions made?
   Probes: shared decisions?

4. How did you move between the various clinicians?
   Probes: who initiated; shared decision?

5. How have you managed your leg pain yourself?
   Probes: what helps, strategies tried

6. What aspects of your management have been most and least helpful?
   Probes: positive/ not so, what was most important/ useful

For peer review only

Fig. 3

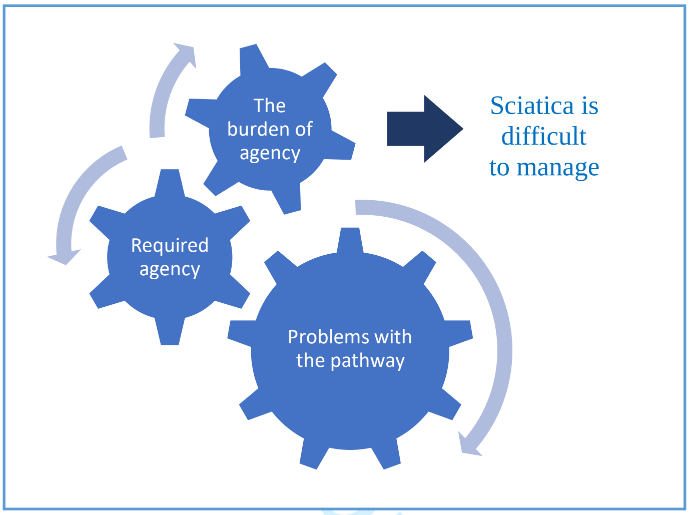

For peer review only

Fig. 4

- Insufficient transparency and information
- Clinician-led decisions
- Standardised care
- Restricted access to specialist care
- A lack of collaboration between services

For peer review only

Consolidated criteria for reporting qualitative studies (COREQ): 32-item checklist

No	Item	Guide questions/description	Alignment Page no.
**Domain 1: Research team and reflexivity**			
Personal Characteristics			
1.	Interviewer/facilitator	Which author/s conducted the interview or focus group?	4
2.	Credentials	What were the researcher's credentials? *E.g. PhD, MD*	4
3.	Occupation	What was their occupation at the time of the study?	4
4.	Gender	Was the researcher male or female?	4
5.	Experience and training	What experience or training did the researcher have?	4
Relationship with participants			

For peer review only

No	Item	Guide questions/description	Alignment Page no.
6.	Relationship established	Was a relationship established prior to study commencement?	4 (how participants were approached and participant exclusion criteria)
7.	Participant knowledge of the interviewer	What did the participants know about the researcher? e.g. *personal goals, reasons for doing the research*	4 (through the participant information sheet)
8.	Interviewer characteristics	What characteristics were reported about the interviewer/facilitator? e.g. *Bias, assumptions, reasons and interests in the research topic*	4 (researcher's occupation, sex, reason for undertaking the study)
**Domain 2: study design**			
Theoretical framework			
9.	Methodological orientation and Theory	What methodological orientation was stated to underpin the study? *e.g. grounded theory, discourse analysis, ethnography, phenomenology, content analysis*	3
Participant selection			

For peer review only

No	Item	Guide questions/description	Alignment Page no.
10.	Sampling	How were participants selected? *e.g. purposive, convenience, consecutive, snowball*	4
11.	Method of approach	How were participants approached? e.g. *face-to-face, telephone, mail, email*	4
12.	Sample size	How many participants were in the study?	5
13.	Non-participation	How many people refused to participate or dropped out? Reasons?	5
Setting			
14.	Setting of data collection	Where was the data collected? e.g. *home, clinic, workplace*	5
15.	Presence of non-participants	Was anyone else present besides the participants and researchers?	4
16.	Description of sample	What are the important characteristics of the sample? *e.g. demographic data, date*	5
Data collection			

For peer review only

No	Item	Guide questions/description	Alignment Page no.
17.	Interview guide	Were questions, prompts, guides provided by the authors? Was it pilot tested?	5
18.	Repeat interviews	Were repeat interviews carried out? If yes, how many?	5
19.	Audio/visual recording	Did the research use audio or visual recording to collect the data?	5
20.	Field notes	Were field notes made during and/or after the interview or focus group?	5
21.	Duration	What was the duration of the interviews or focus group?	5
22.	Data saturation	Was data saturation discussed?	4
23.	Transcripts returned	Were transcripts returned to participants for comment and/or correction?	5
**Domain 3: analysis and findings**			
Data analysis			
24.	Number of data coders	How many data coders coded the data?	5

For peer review only

No	Item	Guide questions/description	Alignment Page no.
25.	Description of the coding tree	Did authors provide a description of the coding tree?	Supplementary file S1
26.	Derivation of themes	Were themes identified in advance or derived from the data?	5
27.	Software	What software, if applicable, was used to manage the data?	5
28.	Participant checking	Did participants provide feedback on the findings?	5
Reporting			
29.	Quotations presented	Were participant quotations presented to illustrate the themes / findings? Was each quotation identified? e.g. *participant number*	8-10
30.	Data and findings consistent	Was there consistency between the data presented and the findings?	8-10
31.	Clarity of major themes	Were major themes clearly presented in the findings?	8-10
32.	Clarity of minor themes	Is there a description of diverse cases or discussion of minor themes?	8-10

For peer review only
